# The Pandemic within a Pandemic: Testing a Sequential Mediation Model to Better Understand Racial/Ethnic Disparities in COVID-19 Preventive Behavior

**DOI:** 10.3390/healthcare9020230

**Published:** 2021-02-20

**Authors:** James A. Roberts, Meredith E. David

**Affiliations:** Hankamer School of Business, Baylor University, Waco, TX 76798, USA; meredith_david@baylor.edu

**Keywords:** COVID-19, race, ethnicity, health disparities, Health Belief Model

## Abstract

Recent Centers for Disease Control and Prevention (CDC) data reveal that COVID-19 hospitalization and mortality rates are higher for certain racial/ethnic groups. Labeled as the “pandemic within a pandemic”, African Americans and Hispanics are bearing more of the brunt of the disease compared to Caucasians. Testing a new sequential mediation model on a sample of 483 US African American, Caucasian, and Hispanic adults, the present study investigates the role of fear of COVID-19, information receptivity, perceived knowledge, and self-efficacy to explain disparities in preventive behaviors. Study contributions include the specification of a new predictive model that improves upon the long-used Health Belief Model (HBM). The Sequential Mediation Model appears to have greater explanatory capacity than the HBM. Study results also provide important insights into racial/ethnic differences in health-seeking behavior related to the coronavirus. Findings show that African Americans reported higher levels of preventive behaviors and self-efficacy than Caucasians. It is possible that SES, rather than race per se, is more important in explaining differences in COVID-19 preventive behaviors. Certain “cues to action” (precipitating factors) also help explain this somewhat surprising result. Additionally, significant differences were found across the three racial/ethnic groups for all the new model’s variables except perceived knowledge. The new model was supported across all three racial/ethnic groups with notable differences across each group. Given the severity of implications surrounding the COVID-19 pandemic (physical, mental, and economic), it is critical that an improved understanding of what drives individual health-seeking behavior be achieved. Study limitations and future research suggestions are discussed.

## 1. Introduction

The COVID-19 pandemic continues to spread both in the U.S. and globally. As of 8 February 2021, there have been 2,671,047 COVID-19 cases diagnosed in the US and 106,810,632 world-wide. In the US, 460,582 people have died and 2,329,906 world-wide as a result of the Coronavirus pandemic [1,2]. Recent data, however, suggests that the ravages of the current pandemic may be worse for certain racial/ethnic groups [3]. Labelled by some as the “pandemic within a pandemic” [4], African Americans and Hispanics are bearing more of the brunt of the disease compared to their Caucasian counterparts [3]. The differential impact of the coronavirus across ethnic groups was brought to light when the first eleven medical doctors in the UK to die from the coronavirus were from BAME (Black, Asian, and Minority ethnic) communities [5].

The historical trend of marginalized ethnic groups being adversely impacted by health crises [6] continues in the US regarding the current COVID-19 pandemic. According to the US Centers for Disease Control and Prevention, the population-wide hospitalization rate for COVID-19 is 102.5 per 10,000 people. In the African American and Hispanic populations, however, the hospitalizations rates are 4.7 and 4.5 times higher than Caucasians, respectively [7].

Mortality rates provide an even clearer picture of the racial disparities of the coronavirus. Research by Gross et al. [3] found that the risk of death from the coronavirus was 3.6 times higher for African Americans and 1.9 times higher for the Hispanic/Latino population compared to non-Hispanic whites. A study by Harvard researchers found that non-Hispanic whites lost 33,446 “years of potential life” compared to 45,777 for African Americans, and 48,704 for Hispanics [8].

The causes for such racial disparities are numerous and interrelated [7,9]. As noted by Hooper et al. [10], the reasons for racial disparities in health outcomes “include social and structural determinants of health, racism, and discrimination, economic and educational disadvantages, health care access and quality, individual behavior, and biology” (p. E1).

None of these factors can be identified as the sole, or even primary factor, for such health disparities; rather, it is the confluence of such factors that result in ethnic minority groups suffering more from health crises. Socioeconomic status, however, plays an important role in explaining disparity in racial health outcomes. Low income individuals are more likely to live in densely populated areas, live in areas surrounded by health risks, are geographically distant from grocery stores and medical facilities, live in multigenerational households where social distancing may be difficult, not have medical insurance or paid sick leave, may work on “front-line” jobs where exposure is high (or be unemployed) and use public transportation [7,11].

In addition to all the reasons above, African American and Hispanic populations also have higher levels of underlying health conditions including hypertension, diabetes, obesity, and cardiovascular disease [7]. Anthony Fauci, a regular figure on US television in the midst of the pandemic and Director of the National Institute for Allergy and Infectious Diseases, has stated that African Americans have a higher incidence of such preexisting conditions that increase the likelihood of suffering from more serious outcomes associated with the coronavirus disease [12]. Mistrust of the health care system and possible language barriers increase the likelihood that at-risk ethnic groups will not pursue medical care for their disease or will not do so until the disease has progressed to the point where more serious negative outcomes of the disease are likely.

Stress also likely plays a role in the more severe negative outcomes of the COVID-19 disease for ethnic populations. “Weathering”—advanced mental and physical aging caused by living in a stressful environment where individuals are confronted with a constant stream of fight-or-flight decisions [13] has been used most often to describe the stressful nature of living in a racially stratified world. Family dysfunction, violent neighborhoods, food insecurity, and the lack of money and other resources all increase the stress levels experienced by many African Americans and Hispanics living in the US.

### Study Objectives

Despite all the potential reasons why health disparities resulting from the COVID-19 pandemic exist, no research to date has investigated the role of individual health-seeking behavior. Building upon the Health Belief Model (HBM) and its many variations, the primary objective of the present study is to identify which variables best predict preventive health behaviors such as social distancing, washing hands, wearing masks, and avoiding large gatherings of people across samples of Caucasians, African Americans, and Hispanics. The present study will identify if variables such as fear of COVID-19, one’s sense of susceptibility to the disease, information receptivity (information seeking), perceived knowledge and self-efficacy are significantly associated with performing COVID-19 preventive behaviors. Even with the recent distribution of a vaccine, the model created for this study can be applied to a wide variety of preventive health behaviors. Additionally, there is little doubt that this will not be the last Coronavirus that will require individual preventive behaviors to reduce its spread.

**Figure 1:**
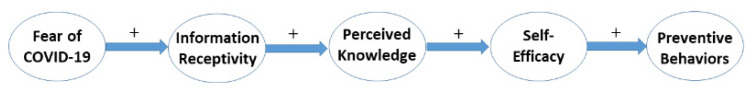
Sequential Mediation Model for Predicting COVID-19 Preventive Behavior.

A second objective of the present study is to identify a more optimal combination of predictor variables and improve the model’s explanatory capacity over earlier attempts to explain health-seeking behavior. Past research has failed to find the best combination of the HBM’s variables which has reduced its ability to explain variations in health-seeking behaviors. This study specifies and finds a combination of variables (see Figure 1) that is more defensible and thus, increases its explanatory capacity and usefulness. Next, we briefly discuss the long history of use of the HBM and describe the variables included in our new sequential mediation model.

## 2. Literature Review

### A New Model for Predicting COVID-19 Preventive Behavior

The Health Belief Model (HBM) has a long history of use in predicting adoption of preventive health behaviors and has been found to be adapted successfully across cultures and contexts [14]. A Google search in June of 2020 of the phrase, “Health Belief Model (Hochaum, Rosenstock and Kegels, 1952)” generated 4590 results. The original version of the HBM was created by Godfrey Hocbaum, Stephen Kegels, and Irwin Rosenstock in the early 1950s. At that time the authors were working for the public health service in the United States.

The HBM was the first theory-based attempt developed solely to explain preventive health behaviors [15]. The model was an attempt to integrate stimulus response theory and social cognitive theory to better explain why individuals did or did not adopt various health-related behaviors [16] ranging from cervical cancer and breast cancer screening, smoking cessation, contraceptive use, stress management, healthy eating, the adoption of exercise regimens, to preventive skin cancer and oral care behaviors to name only a few of its many applications [14,15,17].

The original version of the HBM posited that the likelihood of engaging in a preventive health behavior is a function of four variables: perceived susceptibility of getting the disease, perceived severity (the seriousness or danger associated with catching the disease), perceived benefits of enacting the preventive behaviors, and perceived barriers which make taking the needed actions difficult. Self-efficacy, a person’s assessment of his or her ability to perform the needed behavior, was later added to the model [16]. Cues to actions later added by Rosenstock et al. [16] entail triggers for health behaviors that include both internal and external cues. Examples of internal cues that might lead to adopting certain COVID-19 preventive behaviors include thinking one might have symptoms of the disease or ruminating about the disease. External cues include media exposure (both amount and type), COVID-19 public service campaigns, COVID-19 related behavior among friends and family, and changes in workplace protocol regarding the disease. Socio-demographic measures including age, sex, income, education, and race are often modeled as modifying factors or control variables. Information receptivity, which measures how receptive individuals are to disease-related information and their likelihood of seeking out such information, was later added to the HBM by Manika and Golden [17].

Despite the wide-spread use and empirical support for the HBM as a tool to explain preventive health behavior, it is not without two purported shortcomings [14,15]. First, several research studies have found that the ability of the HBM to explain preventive behaviors is low, with an average R-squared of about 21 percent [15]. A second purported shortcoming of the original HBM and its various extensions is that researchers have not utilized or determined an optimal combination of its most used predictor variables. Many studies simply entered all predictor variables simultaneously into their regression analyses [17]. Only recently have researchers examined alternative relationships between the HBM’s predictor variables (e.g., mediated and/or moderated models) [14,15]. Most recently, research by Roberts and David [18] has identified what appears to be a more optimal, and defensible, model for predicting COVID-19 health-seeking behaviors.

Using a sequential mediation model, Roberts and David [18] found that fear-of-COVID-19 (as a replacement for perceived threat in the original HBM) begins the process of encouraging COVID-19 preventive behaviors. Ahorsu et al. [19] state that a defining characteristic of an infectious disease is fear. These same authors state that fear of a disease is positively associated with its transmission, morbidity, and mortality rate. If an individual does not feel they are susceptible to getting the coronavirus, or if they do not feel that it is a severe danger, they are not fearful and will be less likely to engage in the recommended preventive behaviors [15,17]. A good example in the current pandemic has been the behavior of college students. Likely because they do not feel that they are likely to contract the coronavirus or that it will have severe consequences, U.S. college students congregated on the nation’s beaches at the onset of the coronavirus in March of 2020. The American government and media have attempted to teach young people that they must perform preventive behaviors, not so much for their sake but for the sake of others. Such altruistic behavior, particularly in the United States, will be much more difficult to encourage not only amongst young people but the general population as well [20].

Roberts and David [18] found that those who were more fearful of COVID-19 were more likely to search (information receptivity) for information that could keep them safe from the coronavirus. The more receptive and willing individuals were to search for information related to the coronavirus, the higher their perceived knowledge regarding the disease. Higher levels of perceived knowledge were associated with increased self-efficacy. The more confident an individual is that they can perform the behaviors needed to avoid contracting the coronavirus, the more likely they were to perform the COVID-19 preventive behaviors-social distancing, hand washing, wearing face masks, and avoiding large gatherings. Self-efficacy is regularly found to be the most important predictor of health-related behaviors [15,21]. With the belief that one is equipped with the needed knowledge regarding how to optimally avoid contracting the coronavirus and the confidence that they can effectively engage in these behaviors, the likelihood of performing COVID-19 preventive behaviors was found to increase [18]. R-squared for the Roberts and David [18] full model was 47 percent.

The present study applies the sequential mediation model developed by Roberts and David [18] across three separate racial/ethnic samples: African Americans, Caucasians, and Hispanics. Given the disproportionately negative health outcomes associated with the coronavirus in the US, this study investigates whether such disparities may be a function of differing attitudes, beliefs, and behavior regarding the coronavirus. Since the timetable for finding a cure or, a timely roll-out of the current vaccines for the coronavirus are both highly debatable, it appears that individual preventive behaviors are critical to “flattening the curve” of its spread. Other solutions to mitigate such future health outcome disparities including, for example, affordable healthcare, health insurance for all, and reduced income disparities across racial groups require a much longer-term and collective effort.

## 3. Method

### 3.1. Sample and Procedures

The study involved participants completing an online survey designed using Qualtrics. Individuals ages 18+ living in the U.S. were recruited to participate in the study using Turk Prime with the quota requirement that the final example would include at least 115 participants from each of the three focal ethnic groups. Informed consent forms were completed by all study participants. The study was conducted in June 2020, and all data were collected across a three-week time period beginning on 2 June. A total of 505 individuals participated in the study; 22 respondents’ responses were removed from the data prior to analyses because they either did not pass a quality check or did not respond to the ethnicity/race question. The final sample included a total of 483 participants (55% male, *M*_age_ = 35, *SD* = 1.45). One-third of participants were African American (33%), 43 percent were Caucasian, and 24 percent were Hispanic. Demographic details and descriptive statistics of the sample are shown in Table 1.

### 3.2. Measures

Fear of COVID-19 (α = 0.92) was assessed using the 7-item scale developed and validated by Ahorsu et al. [19] and used recently in related research by Roberts and David [18]. Participants responded to the scale which included items such as, “My hands become clammy when I think about coronavirus-19”, using a 5-point Likert scale. Information receptivity (α = 0.85) was assessed using the 3-item measure used by Roberts and David [18] which was originally adapted from the Manika and Golden [17]. Participants responded to the measure which included items such as, “I actively search for information about the coronavirus,” by indicating their agreement on 7-point scale.

Perceived knowledge (α = 0.77) was measured using an established 4-item scale [17,18]. A 5-point response format anchored with *nothing* (1) and *a lot* (5) was used by participants to respond to the scale which included items such as, “How much do you think you know about the ways a person can and cannot get COVI-19.” Self-efficacy (α = 0.87) was measured with the 5-item scale used by Roberts and David [18] and adapted from the Manika and Golden [17] and Bandura’s [22] measure of self-efficacy. Participants responded to the items, such as “How confident do you feel about your ability to use your knowledge of COVID-19 in making everyday activity choices”, using a 7-point scale anchored with *not at all confident* and *completely confident*.

Prevention behavior (α = 0.84) was assessed using the Manika and Golden [17] measure which included 4-items such as, “I have changed my behavior to try to avoid getting COVID-19”, which were measured with a 7-point Likert scale. At the end of the study, participants responded to demographic questions as well as several cues to action.

## 4. Results

### 4.1. Descriptive Analyses

Since, as reported in Table 2, age, gender, education, and income were correlated with one or more of the focal study variables within and/or across ethnic groups, these demographic characteristics were included as covariates in the analyses presented below.

A Multiple Analysis of Covariance (MANCOVA) was conducted to test whether differences exist in the focal study variables across ethnic groups. Results of the MANCOVA tests of mean differences between ethnic groups reveal significant differences in fear of COVID-19 (F_6, 480_ = 9.289, *R*^2^ = 0.11), information receptivity (F_6, 480_ = 6.464, *R*^2^ = 0.08), self-efficacy (F_6, 480_ = 6.030, *R*^2^ = 0.07), and prevention behaviors (F_6, 480_ = 4.473, *R*^2^ = 0.05). Mean scores of perceived knowledge did not differ across groups (F_6, 480_ = 0.850, *R*^2^ = 0.01). The mean scores of the study variables across ethnic groups are provided in Table 3.

Pairwise comparisons revealed significant differences between Caucasians and Hispanics in fear of COVID-19 (*p* < 0.05), as well as a marginally significant difference between Caucasians and African Americans (*p* = 0.075). Caucasians were particularly fearful of COVID-19, while Hispanics were the least fearful. The mean scores are shown in Table 3. Pairwise comparisons also revealed significant differences in information receptivity between Caucasians and Hispanics (*p* < 0.05) and between African Americans and Hispanics (*p* < 0.05). Caucasians were particularly receptive to information, while Hispanics were less receptive than both Caucasians and African Americans. Self-efficacy scores differed between Caucasians and African Americans (*p* < 0.05), as did preventive behaviors (*p* < 0.05). African Americans were higher in self-efficacy and preventive behavior than Caucasians.

### 4.2. Sequential Mediation Analyses

Next, we tested the Roberts and David [18] conceptual framework which draws from the HBM to predict and explain individuals’ likelihood of engaging in preventive behaviors. We conduct a series of analyses beginning with testing the sequential mediation model using our full dataset, followed by testing the sequential mediation model for each of the three ethnic group samples (African Americans, Caucasians, and Hispanics). We expect to find that, while the overall Roberts and David [18] model is supported, paths in the serial mediation are more nuanced than previously suggested, and these important differences are a function of one’s ethnicity.

To begin, the PROCESS Model 6 [23] was used to test the conceptual model including predictions involving sequential mediation [24] using the full sample inclusive of all ethnic groups. The model first tests the relationship between fear of COVID-19 and information receptivity. The results (F_5, 501_ = 54.61, *p* < 0.01, *R*^2^ = 0.35) indicate that fear of COVID-19 is positively associated with information receptivity (*β* = 0.78, *p* < 0.01).

Next, the model tests whether fear of COVID-19 and information receptivity are directly associated with perceived knowledge. The results (F_6, 500_ = 22.91, *p* < 0.01, *R*^2^ = 0.22) indicate that information receptivity is directly associated with perceived knowledge (*β* = 0.25, *p* < 0.01). However, fear of COVID-19 is not directly associated with perceived knowledge (*p* > 0.05). The model next tests the relationship that fear of COVID-19, information receptivity, and perceived knowledge have with self-efficacy. The results F_7, 499_ = 43.24, *p* < 0.01, *R*^2^ = 0.38) show a significant relationship between fear of COVID-19 and self-efficacy (*β* = −0.17, *p* < 0.05), as well as between perceived knowledge and self-efficacy (*β* = 0.82, *p* < 0.05). Information receptivity is not directly associated with self-efficacy (*p* > 0.05).

In a final step, the model tests whether fear of COVID-19, information receptivity, perceived knowledge, and self-efficacy are directly associated with prevention behaviors. The results F_8, 498_ = 54.09, *p* < 0.01, *R*^2^ = 0.47) indicate that self-efficacy (*β* = 0.49, *p* < 0.01), information receptivity (*β* = 0.30, *p* < 0.01) are directly associated with prevention behaviors. However, fear of COVID-19 and perceived knowledge are not directly associated with prevention behaviors (*p* > 0.05). Importantly, the results show support for sequential mediation (*β* = 0.08; SE = 0.01, 95% CI: 0.06, 0.11), such that fear of COVID-19 is indirectly associated with prevention behaviors via information receptivity, followed by perceived knowledge, and then self-efficacy. A summary of all direct and indirect paths tested in the model is provided in Table 4. Similar results were found when the same model was run but without the demographic control variables.

Next, and as explained above, we ran the same sequential mediation model but on each of the different ethnic group samples. The full sequential mediation model with fear of COVID-19 leading to information receptivity, perceived knowledge, self-efficacy, and ultimately to prevention behavior is significant for all three ethnic groups. However, there are several notable differences in results from the sequential mediation models across ethnic groups, as highlighted in Table 3 and summarized below.

In terms of indirect effects of fear of COVID-19 on preventive behaviors, the results show that among African Americans and Caucasians, fear of COVID-19 indirectly affects prevention behavior through self-efficacy (model 13). This is not the case for Hispanics. Caucasians are the only ethnic group whereby fear of COVID-19 indirectly affects preventive behavior through information receptivity and subsequently, perceived knowledge (model 14). However, Hispanics are the only ethnic group whereby fear of COVID-19 impacts prevention behavior through perceived knowledge and resulting self-efficacy (model 16). Overall, the strongest model of fear of COVID-19 as a driver of prevention behavior across ethnic groups appears to be the simple mediation model in which information receptivity mediates the relationship between fear of COVID-19 and prevention behavior such that fear drives information seeking which fosters preventive behaviors.

## 5. Discussion

The current coronavirus pandemic has had a disproportionately negative impact on the U.S. African American and Hispanic populations compared to Caucasians. The mortality rates are 3.6 times higher for African Americans and 1.9 times higher for Hispanics compared to Caucasians. The present study is the first to investigate the role of individual health-seeking behavior regarding the coronavirus across the three major ethnic groups in the U.S. The study utilizes a sequential mediation model to identify which variables might help us to better understand the behaviors such as social distancing, mask wearing, and hand washing that have been identified as critical to “flattening the curve” of the spread of the coronavirus.

The present study’s results provide important insights, some expected, some not, into ethnic differences in health-seeking behaviors related to the coronavirus. An additional valuable contribution of the present study’s results is that a more optimal ordering of the predictor variables of COVID-19 preventive behavior is identified. The HBM has been in use since the 1950s but has been criticized for not identifying the optimal ordering of its variables and for lower explanatory capacity than desired. The present study, across four samples (full, African American, Caucasian, and Hispanic samples), found that an optimal combination of variables can be captured with a sequential mediation model. Additionally, R-squared for the full model was 0.47 which is a vast improvement over the average R-squared of approximately 0.21 [15] for previous uses of the model. Identifying a more optimal combination of predictor variables and boosting explanatory capacity is critical given the important role individual behavior plays in slowing the spread of the Coronavirus. Such insights will allow health-care professionals, public policy makers, and marketers to better understand what needs to be done to encourage health-seeking behaviors.

Regarding ethnic differences across the three groups, the most surprising result might be that African Americans in the present sample reported higher levels of preventive behaviors and self-efficacy than Caucasians. We expected, given the higher incidence of coronavirus in the general population of African Americans, that the prevalence of preventive behaviors might have been lower among this ethnic group. To better understand this apparent paradox, we conducted a post hoc analysis which investigated whether certain cues to action best understood as precipitating events (e.g., experiencing coronavirus symptoms, knowing someone who has the coronavirus, talking with friends and family about the coronavirus and media exposure, etc.) might help explain why African Americans reported performing more health-seeking behaviors.

Table 5 contains the results of this analysis. As can be seen in Table 5, African Americans were significantly more likely than Hispanics to have friends that are practicing safe behaviors to avoid getting COVID-19 (Cue 3). Friends play an important role in our behavior, and during the present pandemic, friends might even require other friends to practice COVID-safe behaviors if they want to socialize. African Americans were also more likely than Caucasians to report that they have seen or heard “a lot of reminders” (Cue 5) on how to avoid getting COVID-19. This higher level of exposure to COVID-19 messages helps explain why African Americans reported high levels of preventive behaviors and feel confident that they can perform such behaviors (self-efficacy). Additionally, a lot of media coverage has been devoted to the racial disparities in COVID-19 health outcomes. This could have sensitized African Americans to the importance of wearing masks, social distancing, and washing their hands. Although not statistically significant, African Americans reported a stronger agreement with the statement that their workplace has initiated safety measures to avoid contracting COVID-19 (Cue 6). As “front-line” workers, this increased focus on safe behaviors may have carried over into the rest of their lives.

Additionally, the socio-economic status (SES) of African Americans is not statistically different than Caucasians across the present samples. In the general population, the income disparity between the two groups regarding median income is USD 70,642 versus USD 41,361 for Caucasians and African Americans, respectively (https://www.pgpf.org/blog/2019/10/income-and-wealth-in-the-united-states-an-overview-of-data, accessed on 20 February 2021). Past research has shown that SES is an important predictor of health-seeking behaviors [6,25,26]. The SES parity across the two groups might explain the higher than expected self-reported self-efficacy and preventive behaviors in the African American sample.

Additionally, as shown in Table 4, Caucasians were more likely to have contracted COVID-19 than expected (Cue 7). Although not a significant difference, 41 percent of Caucasians (vs. 33 percent of African Americans) reported knowing someone who has had COVID-19 (Cue 8). Both cues are likely drivers of COVID-19 preventive behaviors. However, it is important to note that the relationship between these variables, i.e., preventive behaviors and contracting or knowing someone who has contracted coronavirus, is likely bidirectional. Indeed, it may well be that the less frequent preventive behavior observed among Caucasians in the present study could explain the higher infection rates reported by these individuals.

If the present non-random sample of African Americans is indicative of typical individuals of this ethnic group, why does higher self-efficacy and preventive behaviors still lead to much higher hospitalization and mortality rates in the larger African American population? As mentioned earlier in this article, individual behavior is important, but it is not the only factor that influences hospitalization and mortality rates from COVID-19. Economic and educational disadvantages, lack of access to healthcare and medical insurance, and a plethora of pre-existing conditions can mitigate the benefits of health-seeking preventive behaviors at the individual level.

The first series of analyses included MANCOVA tests of mean differences across each ethnic group for the five variables of the HBM: fear-of-COVID-19, information receptivity, perceived knowledge, self-efficacy, and prevention behaviors. Significant differences were found across all the HBM variables except for perceived knowledge. All groups averaged 3.7–3.8 on a five-point scale of perceived knowledge of the coronavirus and how to avoid it. It appears that most respondents from all ethnic groups believed they were knowledgeable and knew more (vs. less) regarding the coronavirus (as compared to the scale midpoint of 3). Although this is a self-assessment of perceived knowledge, rather than a measure of actual knowledge, the typical respondent feels they have the needed information to avoid contracting the coronavirus. Knowledge, however, whether perceived or actual, is not necessarily a good predictor of the desired health behavior [21]. In fact, the sequential mediation model tested on the full sample found that perceived knowledge was not significantly associated with COVID-19 preventive behaviors.

Interestingly, Caucasians were found to be more fearful of COVID-19 than both African Americans and Hispanics, the latter of which who were least afraid of the disease. This, in turn, led to lower information receptivity on the part of Hispanics. Hispanic respondents were less receptive and less likely to search for COVID-related information. In contrast, Caucasians searched for more information than both Hispanics and African Americans.

The sequential mediation model tested on the full sample found that fear-of-COVID-19 and perceived knowledge were not significantly associated with COVID-19 preventive behaviors. Information receptivity and self-efficacy were significantly associated with COVID-19 preventive behaviors and explained 47 percent of such behaviors. This level of R-squared is encouraging but suggests that 53 percent of variation in COVID-19 preventive behaviors remains unexplained. The myriad of social and structural constraints that impact health outcomes can interfere with the performance of the needed health behaviors. For example, social distancing might be impossible in multigenerational or overcrowded households. Masks, wipes, and sanitizing hand gel may not be easily available or afforded.

The full sequential mediation model (see Figure 1) leading to COVID-19 preventive behaviors was significant across all three ethnic groups with several notable differences across each ethnic group. Fear-of-COVID-19 had an indirect effect on preventive behaviors through self-efficacy only for African Americans and Caucasians. For Hispanics, fear-of-COVID-19 indirectly affected prevention behaviors mediated by both perceived knowledge and self-efficacy sequentially. For Caucasians, fear-of COVID-19 indirectly affected preventive behaviors through information receptivity and perceived knowledge. Results show that the best model of fear-of-COVID-19 as a driver of preventive behavior is through its impact on information receptivity. Fear of COVID-19 motivates people to attend to, and search for, COVID-19 related information. Such receptivity then leads directly to COVID-19 preventive behaviors. Given that the needed behaviors to protect oneself from COVID-19 are easy to understand and enact once individuals have such information, they are more likely to perform the needed behaviors.

## 6. Limitations and Future Research Directions

Although this study is the first to investigate racial disparities in COVID-19 health-seeking behavior, its results must be tempered by certain limitations. Three separate samples were collected across the three largest ethnic/racial groups in the U.S., but larger, random samples are needed to aid generalization of the study’s results. The primary focus of this study was to identify which variables (and the optimal combination of variables) were most helpful in explaining COVID-19 related health-seeking behaviors across the three racial/ethnic groups. However, given the limitations of our non-random samples (from individuals ages 18+ who live in the U.S.), the findings presented herein cannot be assumed to generalize to the overall African American, Caucasian, and Hispanic populations in the U.S. or across the globe.

A second area of future research should focus on continued improvement of our ability to understand individual health-seeking behavior. Although the present study identified what appears to be an optimal, and defensible, combination of predictor variables, further research is needed. The role of cues to action remain to be properly specified within the new model. A valid scale of such cues would likely improve the model’s explanatory capacity. Jones et al. [14] suggest that cues to action might better be broken down into internal cues to action such as perceived COVID-19 symptoms or ruminating about the disease and external cues to action such as media exposure or COVID-related workplace requirements. The authors further suggest it might be enlightening to investigate manipulated cues to action like PSAs, media messages, and interventions and more “organic” cues to action like COVID-19 illness among friends or family members, and celebrities or other high-profile individuals who contract the disease. The inclusion of such cues to action will likely boost the new model’s explanatory ability but, to date, have only been used in models where all the HBM variables are entered into the analytical model simultaneously. It might be that both fear of COVID-19 and cues to action impact an individual’s information receptivity. Or cues to action may be associated with one’s fear of COVID-19. Further research is needed to address such issues.

Lastly, other models used to predict COVID-19 health-seeking behaviors must be tested alongside the sequential mediation model. Research by Taylor et al. [26] compared the HBM with the Theory of Reasoned Action, the Theory of Planned Behavior, and the Trans-Theoretical Model in predicting health-related behavioral change. The authors note each model has its own unique aspects. Results of their research suggest that further testing of the efficacy of each model to predict health-seeking behaviors is needed. This is particularly true given the greater availability of information (misinformation) provided by the Internet, Smartphones, and other technologies. Additionally, as in the present study, all models must be tested for culturally sensitivity.

Given the severity of implications from the COVID-19 pandemic (physical, mental, and economic), it is critical that an improved understanding of what drives individual health-seeking behavior be achieved. The fact that the disease disproportionately impacts certain segments of U.S. society more than others compels us as a moral society to do all that is necessary to stop its spread amongst all people. Former US Vice-President, Hubert H. Humphrey, said in 1977, “the moral test of government is how that government treats those who are in the dawn of life, the children; those who are in the twilight of life, the elderly; those who are in the shadow of life; the sick, the needy and the handicapped” [27].

## Figures and Tables

**Table 1:** Demographics across Ethnic Groups.

Demographics	African Americans	Caucasians	Hispanics
Age: Mean (range)	36 (10–77)	37 (18–74)	35 (18–69)
Occupational Prestige: Mean (SD)	17.25 (5.38)	17.39 (4.79)	15.79 (5.72)
Gender:			
Female	48%	41%	47%
Male	52%	59%	53%
Education:			
Highschool or Less	4%	11%	15%
Some College	21%	12%	18%
Associate degree	14%	7%	11%
Bachelor’s Degree	44%	54%	43%
Master’s Degree	14%	14%	10%
Doctoral Degree	3%	2%	3%
Income:			
Less than USD 20 k	7%	14%	12%
USD 20 k-USD 39,999	23%	20%	29%
USD 40 k-USD 59,999	32%	26%	24%
USD 60 k-USD 79,999	21%	19%	10%
USD 80 k-USD 99,999	7%	8%	11%
USD 100 k or more	10%	13%	14%

**Table 2:** Demographic Variables’ Correlation Coefficients with Focal Study Measures.

Demographics	Fear of COVID-19	Information Receptivity	Perceived Knowledge	Self-Efficacy	Prevention Behaviors
Age	−0.134 −0.035 0.017	−0.062 −0.038 0.092	0.069 −0.022 0.136	0.161 * 0.148 * 0.110	0.152 0.142 * 0.144
Gender	−0.176 * −0.170 * 0.077	−0.146 −0.171 * −0.049	−0.026 −0.039 0.012	0.212 ** 0.133 0.114	0.089 0.030 0.006
Education	0.212 ** 0.114 0.099	0.213 ** 0.121 0.144	0.074 −0.049 0.041	−0.067 −0.121 0.020	0.019 −0.078 0.144
Income	−0.079 −0.214 ** −0.129	0.069 −0.055 −0.055	0.098 0.035 −0.092	0.113 0.082 0.002	0.169 * 0.079 0.034

Note. * *p* < 0.05, ** *p* < 0.01. Row 1 = African Americans, Row 2 = Caucasians, Row 3 = Hispanics.

**Table 3:** Means, Standard Deviations, and Correlation Coefficients for Focal Study Variables.

Study Variables	M	SD	Fear of COVID-19	Information Receptivity	Perceived Knowledge	Self-Efficacy
Fear of COVID-19	3.00 3.26 2.71	1.08 1.05 1.05				
Information Receptivity	4.84 5.01 4.30	1.51 1.31 1.51	0.630 ** 0.530 ** 0.508 **			
Perceived Knowledge	3.82 3.78 3.67	0.66 0.66 0.76	0.256 ** 0.231 ** −0.021	0.459 ** 0.573 ** 0.349 **		
Self-Efficacy	5.88 5.52 5.67	0.96 1.00 1.12	−0.031 −0.059 −0.098	0.210 ** 0.272 ** 0.129	0.452 ** 0.666 ** 0.547 **	
Prevention Behavior	5.77 5.41 5.51	1.03 1.05 1.22	0.233 ** 0.235 ** 0.240 **	0.511 ** 0.559 ** 0.422 **	0.460 ** 0.581 ** 0.271 **	0.580 ** 0.592 ** 0.415 **

Note. ** *p* < 0.01. Row 1 = African Americans, Row 2 = Caucasians, Row 3 = Hispanics.

**Table 4:** Sequential Mediation Results for All Models Including All Direct and Indirect Effects.

Study Models	Coefficient	SE	95% LLCI	95% ULCI
Model 1				
Fear of COVID-19 → Information Receptivity	**0.78**	0.09	0.69	0.89
African Americans	**0.88**	0.09	0.70	1.06
Caucasians	**0.65**	0.04	0.50	0.80
Hispanics	**0.74**	0.12	0.50	0.98
Model 2				
Fear of COVID-19 → Perceived Knowledge	**−0.08**	0.03	−0.15	−0.02
African Americans	−0.01	0.06	−0.13	0.10
Caucasians	−0.06	0.04	−0.14	0.03
Hispanics	**−0.20**	0.08	−0.35	−0.05
Model 3				
Information Receptivity → Perceived Knowledge	**0.25**	0.02	0.20	0.29
African Americans	**0.21**	0.04	0.13	0.19
Caucasians	**0.33**	0.03	0.26	0.40
Hispanics	**0.24**	0.05	0.14	0.34
Model 4				
Fear of COVID-19 → Self-Efficacy	**−0.17**	0.04	−0.25	−0.09
African Americans	**−0.16**	0.08	−0.32	−0.01
Caucasians	**−0.16**	0.07	−0.27	−0.05
Hispanics	−0.07	0.10	−0.28	0.13
Model 5				
Information Receptivity → Self-Efficacy	0.06	0.03	−0.01	0.12
African Americans	**0.12**	0.08	−0.32	−0.01
Caucasians	−0.02	0.05	−0.12	0.09
Hispanics	−0.02	0.10	−0.28	0.13
Model 6				
Perceived Knowledge → Self-Efficacy	**0.82**	0.06	0.71	0.94
African Americans	**0.61**	0.11	0.38	0.83
Caucasians	**1.08**	0.09	0.90	1.26
Hispanics	**0.80**	0.13	0.55	1.06
Model 7				
Fear of COVID-19 → Prevention Behaviors	0.07	0.04	−0.02	0.16
African Americans	0.02	0.07	−0.12	0.16
Caucasians	0.08	0.06	−0.04	0.20
Hispanics	0.13	0.11	−0.10	0.35
Model 8				
Information Receptivity → Prevention Behaviors	**0.30**	0.03	0.23	0.37
African Americans	**0.27**	0.06	0.16	0.38
Caucasians	**0.32**	0.06	0.21	0.43
Hispanics	**0.26**	0.08	0.10	0.43
Model 9				
Perceived Knowledge → Prevention Behaviors	−0.03	0.07	−0.17	0.11
African Americans	0.11	0.11	−0.10	0.32
Caucasians	0.08	0.12	−0.16	0.33
Hispanics	−0.12	0.16	−0.44	0.21
Model 10				
Self-Efficacy → Prevention Behaviors	**0.49**	0.05	0.40	0.58
African Americans	**0.46**	0.07	0.31	0.60
Caucasians	**0.44**	0.07	0.29	0.58
Hispanics	**0.45**	0.10	0.25	0.66
Model 11				
Fear of COVID-19 → Information Receptivity → Prevention Behavior	**0.23**	0.04	0.16	0.32
African Americans	**0.23**	0.07	0.12	0.37
Caucasians	**0.21**	0.07	0.08	0.37
Hispanics	**0.20**	0.05	0.10	0.31
Model 12				
Fear of COVID-19 → Perceived Knowledge → Prevention Behavior	<0.01	0.01	−0.01	0.02
African Americans	−0.01	0.01	−0.02	0.02
Caucasians	−0.01	0.01	−0.03	0.03
Hispanics	0.02	0.04	−0.05	0.12
Model 13				
Fear of COVID-19 → Self-Efficacy → Prevention Behavior	**−0.08**	0.02	−0.13	−0.04
African Americans	**−0.08**	0.04	−0.15	−0.01
Caucasians	**−0.07**	0.03	−0.14	−0.01
Hispanics	−0.03	0.05	−0.14	0.07
Model 14				
Fear of COVID-19 → Information Receptivity → Perceived Knowledge → Prevention Behavior	−0.01	0.02	−0.04	0.03
African Americans	0.02	0.02	−0.02	0.07
Caucasians	**0.02**	0.03	−0.14	−0.01
Hispanics	−0.02	0.03	−0.10	0.04
Model 15				
Fear of COVID-19 → Information Receptivity → Self-Efficacy → Prevention Behavior	0.02	0.02	−0.01	0.05
African Americans	0.05	0.03	−0.01	0.10
Caucasians	−0.01	0.04	−0.06	0.10
Hispanics	−0.01	0.03	−0.06	0.05
Model 16				
Fear of COVID-19 → Perceived Knowledge → Self-Efficacy → Prevention Behavior	**−0.04**	0.01	−0.06	−0.01
African Americans	−0.01	0.02	−0.05	0.03
Caucasians	−0.03	−0.03	−0.08	0.02
Hispanics	**−0.07**	0.04	−0.16	−0.02
Model 17				
Fear of COVID-19 → Information Receptivity → Perceived Knowledge → Self-Efficacy → Prevention Behavior	**0.08**	0.01	0.06	0.11
African Americans	**0.05**	0.02	0.02	0.10
Caucasians	**0.10**	0.03	0.05	0.15
Hispanics	**0.07**	0.03	0.02	0.13

Note. Results obtained with bootstrapping (*n* = 5000); age, gender, education, and income range were included as covariates; the results did not differ when no covariates were included in the model. The top row within each cell corresponds to results from the full sample; the second-row reports results from the African American sample, etc. Coefficients in bold denote significant effects (*p* < 0.05). Model results from the analyses predicting information receptivity for each ethnic group are as follows: African Americans: F_5, 155_ = 22.00, *p* < 0.01, *R*^2^ = 0.42; Caucasians: F_5, 199_ = 16.38, *p* < 0.01, *R*^2^ = 0.29; Hispanics: F_5, 109_ = 8.46, *p* < 0.01, *R*^2^ = 0.28. Model results from the sequential mediation analyses predicting perceived knowledge for each ethnic group are as follows: African Americans: F_6, 154_ = 7.48, *p* < 0.01, *R*^2^ = 0.23; Caucasians: F_6, 198_ = 18.51, *p* < 0.01, *R*^2^ = 0.36; Hispanics: F_5, 108_ = 4.27, *p* < 0.01, *R*^2^ = 0.19. Model results from the sequential mediation analyses predicting self-efficacy for each ethnic group are as follows: African Americans: F_7, 153_ = 9.27, *p* < 0.01, *R*^2^ = 0.30; Caucasians: F_7, 197_ = 30.77, *p* < 0.01, *R*^2^ = 0.52; Hispanics: F_7, 107_ = 7.32, *p* < 0.01, *R*^2^ = 0.32. Model results from the sequential mediation analyses predicting preventive behaviors for each ethnic group are as follows: African Americans: F_8, 152_ = 20.50, *p* < 0.01, *R*^2^ = 0.52; Caucasians: F_8, 196_ = 29.67, *p* < 0.01, *R*^2^ = 0.55; Hispanics: F_8, 106_ = 6.82, *p* < 0.01, *R*^2^ = 0.34.

**Table 5:** Cues to Action across Ethnic Groups.

Cues to Action	African Americans	Caucasians	Hispanics
Cue1: I have experienced symptoms I thought might be COVID-19	2.53 (1.48)	2.91 (1.38)	2.39 (1.36)
Cue2: I think a lot about COVID-19	3.39 (1.37)	3.60 (1.14)	3.09 (1.35)
Cue3: My friends are practicing safe behavior to avoid getting COVID-19	4.02 (.98)	3.91 (0.92)	3.69 (1.03)
Cue4: My job situation has changed because of COVID-19	3.78 (1.36)	3.51 (1.37)	3.49 (1.45)
Cue5: I have seen or heard a lot of reminders on how to avoid getting COVID-19	4.39 (0.81)	4.07 (0.95)	4.21 (0.89)
Cue6: My workplace has initiated safety measures to avoid contracting COVID-19	4.10 (0.98)	3.87 (.98)	3.85 (1.12)
Cue7: Have/Have had COVID-19	12%	20%	7%

Note: Mean scores are shown with standard deviation in parentheses. Five of the cues to action have significant differences across ethnic groups. For Cue 1, Caucasians are sig. different from African Americans and Hispanics. For Cue 2, Caucasians and Hispanics are sig. different from each other. For Cue 3, African Americans and Hispanics are sig. different from each other. For Cue 5, Caucasians and African Americans are sig. different from each other. For Cue 7, Crosstab/Chi-square analyses show that Caucasians are more likely than expected to have had COVID-19, and Hispanics are less likely than expected to have had it.

## Data Availability

The data presented in this study are available on request from the corresponding author. The data are not publicly available due to the promise to respondents that all data will be stored on password-protected computers.
